# Clinical and electrophysiological features of post-traumatic Guillain-Barré syndrome

**DOI:** 10.1186/s12883-017-0919-x

**Published:** 2017-07-27

**Authors:** Xiaowen Li, Jinting Xiao, Yanan Ding, Jing Xu, Chuanxia Li, Yating He, Hui Zhai, Bingdi Xie, Junwei Hao

**Affiliations:** 1 Department of Neurology and Tianjin Neurological Institute, Tianjin Medical University General Hospital, Tianjin, 300052 China; 2 Department of Neurology, Tianjin Haihe Hospital, Tianjin, 300060 China

**Keywords:** Post-traumatic GBS, GBS, Trauma, Electrophysiology, Axonal damage

## Abstract

**Background:**

Post-traumatic Guillain-Barré syndrome (GBS) is a rarely described potentially life-threatening cause of weakness. We sought to elucidate the clinical features and electrophysiological patterns of post-traumatic GBS as an aid to diagnosis.

**Methods:**

We retrospectively studied six patients diagnosed with post-traumatic GBS between 2014 and 2016 at Tianjin Medical University General Hospital, China. Clinical features, serum analysis, lumbar puncture results, electrophysiological examinations, and prognosis were assessed.

**Results:**

All six patients had different degrees of muscular atrophy at nadir and in two, respiratory muscles were involved. Five also had damaged cranial nerves and four of these had serum antibodies against gangliosides. The most common electrophysiological findings were relatively normal distal latency, prominent reduction of compound muscle action potential amplitude, and absence of F-waves, which are consistent with an axonal form of GBS.

**Conclusions:**

It is often overlooked that GBS can be triggered by non-infectious factors such as trauma and its short-term prognosis is poor. Therefore, it is important to analyze the clinical and electrophysiological features of GBS after trauma. Here we have shown that electrophysiological evaluations are helpful for diagnosing post-traumatic GBS. Early diagnosis may support appropriate treatment to help prevent morbidity and improve prognosis.

## Background

Guillain-Barré syndrome (GBS) is a multifactorial and lethal inflammatory demyelinating polyradiculopathy and polyneuropathy, characterized by flaccid paralysis and acute demyelinating changes in the peripheral nervous system [1, 2]. Although a range of infectious factors, such as *Campylobacter jejuni* or cytomegalovirus, are associated with this syndrome, GBS has also been reported to be triggered by non-infectious factors such as trauma [3–6].

Trauma is defined as any physical damage to the body caused by violence or accident. The concept of post-traumatic GBS was recently introduced and defined as GBS preceded by no risk factors other than trauma [4]. To date, there appears to have been no systematic analysis of the clinical and electrophysiological features of GBS following trauma. Therefore, here we performed retrospective analyses to investigate those features.

## Methods

### Subjects

Six patients with GBS that occurred after trauma resulting from surgery or injury were diagnosed in our Department of Neurology between January 2014 and January 2016. All patients in this study met the clinical criteria for GBS (Table 1) [1, 7, 8] and had no risk factors other than trauma. Exclusion criteria for patient selection included a history of prodromal immunization or antecedent infections and prior use of neuromuscular blocking agents or intravenous gangliosides. We performed a retrospective analysis of these six patients’ clinical records in our GBS database reviewing their basic characteristics, neurologic status, serum antibodies against gangliosides, reports of cerebrospinal fluid (CSF) analyses, and electrophysiological data. Because of the retrospective nature of the study, there were no further nerve conduction studies (NCS) or CSF examinations other than those performed at diagnosis.

**Table 1:** Diagnosis of GBS

Features required for diagnosis	
Progressive weakness in both arms and legs (might start with weakness only in the legs)	
Areflexia (or decreased tendon reflexes)	
Features that strongly support diagnosis	
Progression of symptoms over days to 4 weeks	
Relative symmetry of symptoms	
Mild sensory symptoms or signs	
Cranial nerve involvement, especially bilateral weakness of facial muscles	
Autonomic dysfunction Pain (often present)	
High concentration of protein in CSF	
Typical electrodiagnostic features	
AMAN	
None of the features of AIDP except one demyelinating feature allowed in one nerve if dCMAP <10% LLN	
Sensory action potential amplitudes normal	
AMSAN	
None of the features of AIDP except one demyelinating feature allowed in one nerve if dCMAP < 10% LLN	
Sensory action potential amplitudes < LLN	

*dCMAP* = compound muscle action potential amplitude after distal stimulation; *LLN* = lower limit of normal

### Evaluation of functional impairment

The clinical severity of the patients’ GBS and their neurologic status were evaluated by calculating their Hughes Functional Grading Scale (HFGS) and Medical Research Council (MRC) sum scores [9, 10]. The nadir of disease was defined as the highest HFGS score or the lowest MRC sum score. The therapeutic efficacy was assessed by the improvement in HFGS and MRC sum scores between nadir and 2 weeks after treatment. All cases were followed up.

### Electrophysiological study

Electrodiagnoses were made using Viking Quest (EMG & Evoked Potential Response Unit, Nicolet, NE, USA), the standard method at our institute. Electrophysiological examinations included NCS and F-wave assessments, which all patients underwent 10–14 days after the beginning of symptoms [11, 12]. Limb temperature was maintained above 32 °C with a heater, if needed. Using surface electrodes and a stimulator for NCS, we performed motor and orthodromic sensory NCS in eight nerves of the bilateral upper and lower extremities (median, ulnar, tibial, and peroneal nerve). In motor nerves, distal latency (DL), amplitude of compound muscle action potential (CMAP), and motor nerve conduction velocity (MCV) were measured. Amplitude of sensory nerve action potential (SNAP) and sensory nerve conduction velocity (SCV) were also evaluated. The incidence of F-waves was measured after 20 supramaximal stimulations of motor nerves (median, ulnar, and tibial nerves). Abnormality was defined as values falling outside the mean ± 2.5 standard deviations of our laboratory control. Diagnosis of axonal or demyelinating neuropathy was based on the electrophysiological criteria proposed by Hadden and colleagues [1].

### Anti-ganglioside antibody assay

Sera from all patients except patient #5 were examined for anti-ganglioside antibodies by enzyme-linked immunosorbent assay (ELISA) at the acute phase of GBS [11, 13, 14]. The ganglioside antigens used in the ELISA were 200 ng each of GM1, GD1b and GQ1b. Only IgG antibodies were considered pathological in this study.

## Results

### Characteristics of the patients

The clinical features of the patients are summarized in Table 1. The mean age was 42.5 years (range 29–57 years), and the group included four women and two men. No complications had occurred following the trauma and all patients were alert and oriented, with stable vital signs and without focal neurological deficits, before the first symptoms of GBS occurred. Patient #2 was admitted to another hospital with a closed head injury after falling. The results of cranial CT imaging and magnetic resonance imaging were normal. Patient #4 was admitted with a rib fracture following an accident. The results of chest CT revealed that the lung appeared normal. Patient #6 was admitted with a femoral fracture after a traffic accident.

**Fig. 1:**
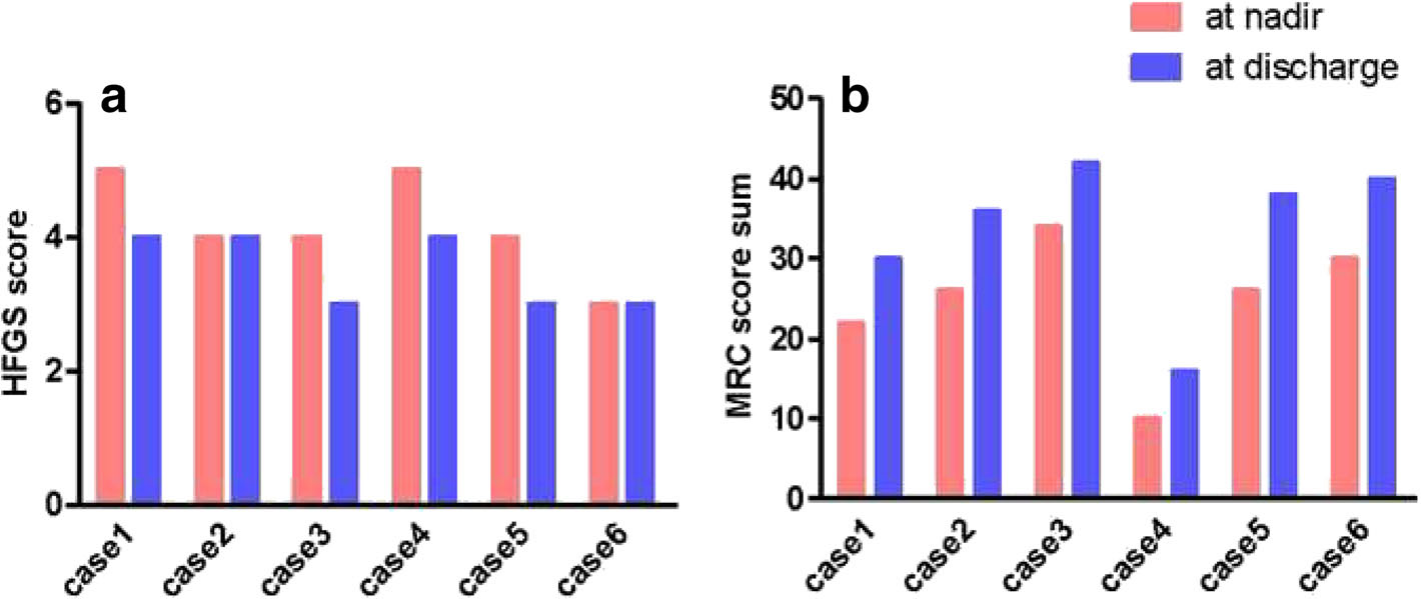
Neurologic status of patients with post-traumatic GBS. **a** Scores of the Hughes Functional Grading Scale (HFGS) were significantly increased in patients compared to normal values, both at nadir and at discharge. This suggests more severe clinical courses and poorer short-term outcomes. **b** The Medical Research Council sum scores (MRC) were significantly decreased in these patients both at nadir and at discharge

The average interval between trauma and the onset of GBS symptoms ranged from 8 to 14 days (average of 11.3 days). However, during the following 7–10 days, the symptoms rapidly worsened. Approximately 2 weeks after GBS onset, all patients underwent lumbar puncture with albumino-cytological dissociation. The principal clinical presentation was progressive symmetrical weakness with varying degrees of muscle atrophy, especially in the lower limbs, and hyporeflexia or areflexia. Two of the six patients had numbness of limbs (#3, #6). Five of the six patients exhibited cranial nerve involvement, and most cranial nerves became affected, generally by palsy of the oculomotor and trochlear nerves (#1, #2, #3, #6), followed by abducens (#1, #2, #3) and vagus nerve deficits (#4). Moreover, the incidence of respiratory muscle paralysis was high, as particularly evident in patients #1 and #4, who required mechanical support for breathing (Table 2). HFGS and MRC scores were also used to evaluate clinical severity and were 4.17 ± 0.75 and 24.67 ± 8.27 at nadir, respectively (Fig. 1).

**Table 2:** Characteristics and clinical presentations of six patients with GBS

Characteristic	Case 1	Case 2	Case 3	Case 4	Case 5	Case 6
Age (y)/Sex	29/F	48/F	29/F	57/M	53/F	39/M
Antecedent events	Abortion	mild Traumatic brain injury	Cesarean section	Chest trauma	Endoscopic endonasal resection of Rathke cyst	Femoral fracture
Time between trauma and symptom onset (days)	14	10	8	10	12	12
Time between treatment initiation and symptom onset (days)	6	5	6	11	9	5
Time to nadir (days)	9	7	10	5	12	7
Time to discharge (days)	33	43	21	56	38	22
Symptoms at nadir						
Motor function	Weakness on both limbs (G2/5)	Weakness on both limbs (G2/5)	Weakness on both limbs (G3/5)	Weakness on both limbs (G1/5)	Weakness on both limbs (G2/5)	Weakness on both limbs (G3/5)
Deep tendon reflexes	Absent (G —)	Absent (G —)	Decreased (G1+)	Absent (G —)	Absent (G —)	Decreased (G1+)
Muscular atrophy at nadir	+	+	+	+	+	+
Cranial nerve function	III, IV, VI, VII	II, III, IV, VI	III, IV, VI	V, IX, X	−	III, IV
Respiratory muscle involvement	−	+	−	+	−	−
Objective sensory function	Normal	Normal	Abnormal	Normal	Normal	Abormal
Serum anti-ganglioside antibody	GQ1b	GM1	GM1,GD1b	−	Missing	GM1,GD1b
Protein (g/L)/AD in CSF	0.98/yes	0.64/yes	1.10/yes	0.92/yes	0.54/yes	0.72/yes
Treatment	IVIG	IVIG; MV	IVIG	IVIG; HC; MV	IVIG	IVIG

*GBS* Guillain-Barré syndrome, *AD* Albumino-cytological dissociation, *IVIG* Intravenous Immunoglobulin, *MV* Mechanical ventilation, *HC* high-dose corticosteroids

### Electrophysiological features

Table 3 shows the patients’ electrophysiological features. The mean interval between the time of NCS and the onset of symptoms was 8.5 (range 6–10) days. Abnormalities were clearly more frequent in motor than sensory nerves. In motor nerves, CMAP amplitude reduction was prominent, and unexcitable nerves were more common in lower than upper limbs. DL and NCV were normal or slightly abnormal in motor nerves. The reduction of CMAP amplitudes was more severe than the slowing of motor conduction. In sensory nerves, SNAP amplitude was relatively preserved in both the upper and lower limbs, and remained normal in some patients (#2, #3, #4). In contrast, all subjects had F-wave abnormalities, the most common of which was reduced F-wave persistence. That is, 62% of examined nerves manifested deleterious F-wave changes, especially in the ulnar nerve.

**Table 3:** Electrophysiological findings of enrolled patients with post-traumatic GBS

		Case 1		Case 2		Case 3		Case 4		Case 5		Case 6	
		L	R	L	R	L	R	L	R	L	R	L	R
CMAP(mV)	Median nerve	2.0	3.5	0.4	2.6	2.2	3.2	2.4	1.2	--	--	2.5	3.4
	Ulnar nerve	1.1	1.8	1.9	3.2	2.0	2.6	1.9	1.2	--	--	1.6	2.8
	Tibial nerve	1.5	2.1	0.8	0.6	1.5	3.2	1.0	1.0	--	--	--	--
	Peroneal nerve	1.2	1.1	1.2	1.0	2.1	2.0	0.6	0.4	--	--	--	--
MCV(m/s)	Median nerve	49	45	65	60	62	58	55	57	--	--	58	50
	Ulnar nerve	50	46	60	56	61	64	52	56	--	--	47	49
	Tibial nerve	44	43	44	43	42	45	50	47	--	--	--	--
	Peroneal nerve	44	44	40	42	41	41	46	45	--	--	--	--
DL(ms)	Median nerve	3.7	3.8	3.8	3.5	3.9	3.3	3.6	3.8	--	--	2.7	3.2
	Ulnar nerve	3.2	3.1	3.8	3.6	2.8	2.9	3.0	2.9	--	--	3.2	2.9
	Tibial nerve	6.2	5.3	5.1	5.1	4.3	4.0	4.3	4.5	--	--	--	--
	Peroneal nerve	4.6	4.4	4.9	5.0	4.1	4.3	4.6	4.3	--	--	--	--
SNAP(uV)	Median nerve	6.3	8.3	15.3	13.5	15.5	13.6	12.6	11.7	11.0	12.5	8.7	10.3
	Ulnar nerve	4.2	6.3	22.6	20.5	14.3	11.5	10.2	11.0	9.2	8.5	7.3	8.2
	Tibial nerve	8.5	8.2	22.1	19.3	12.4	10.6	15.3	11.5	6.3	7.4	8.5	8.7
	Peroneal nerve	12.4	15.3	23.6	20.2	15.8	17.3	21.6	24.3	19.6	15.8	14.7	17.2
SCV(m/s)	Median nerve	59	58	63	60	57	53	51	50	59	56	52	56
	Ulnar nerve	57	55	64	59	62	58	55	51	52	54	55	59
	Tibial nerve	59	59	50	53	56	53	50	49	55	53	50	56
	Peroneal nerve	52	57	53	55	57	52	50	51	54	51	52	57
F-wave(%)	Median nerve	25.0	35.0	0.0	0.0	35.0	30.0	35.0	15.0	--	--	20.0	15.0
	Ulnar nerve	0.0	20.0	0.0	0.0	60.0	45.0	15.0	0.0	--	--	0.0	0.0
	Tibial nerve	0.0	0.0	0.0	0.0	20.0	30.0	30.0	0.0	--	--	--	--

*GBS* Guillain-Barré syndrome, *L* left, *R* right, *CMAP* compound muscle action potential, *MCV* motor nerve conduction velocity, *DL* distal latency, *SNAP* sensory nerve action potential, *SCV* sensory nerve conduction velocity, −− disappearance

### Anti-ganglioside antibodies

Positivity for anti-ganglioside antibodies was detected in sera from five of the six patients (patient #5 refused the examination). IgG antibodies were also present in four patients: the target antigens were GM1 in patients #2, #3 and #6, GD1b in patients #3 and #6, and GQ1b in patient #1.

### Treatment and outcomes

Once GBS was confirmed, treatment with intravenous human immunoglobulin (and a large dose of corticosteroids in patient #4) was performed at a dose of 0.4 g/kg for 5 days. Although this treatment provided clinical improvement, recovery was incomplete, and the outcomes were poor. All patients suffered muscular atrophy, which was apparent to different extents at nadir. High HFGS and low MRC scores were noted both at nadir and at discharge, as shown in Fig. 1.

## Discussion

Post-trauma inflammatory neuropathy, including focal neuropathies, multifocal neuropathy, and diffuse polyneuropathy, was recently defined as neurologic deterioration occurring during the early post-traumatic period [15]. GBS is one such neuropathy that is a rare but severe neurologic complication after trauma. Duncan and colleagues described in 1987 the first identified case of post-traumatic GBS [16]. During the past few decades, several reports have described patients presenting with GBS after multiple types of trauma (Table 2) [15, 17–22]. The requirement for establishing a temporal relationship between a traumatic event and subsequent neuropathy is that the neuropathic symptoms must start within 30 days of the trauma. In the six GBS patients described here, no risk factors other than trauma were identified, and the average interval between trauma and the onset of GBS symptoms ranged from 8 to 14 days (average of 11.3 days). Interestingly, most patients in our study exhibited motor dysfunction with muscular atrophy, significant cranial nerve deficits, and worsening paresis resulting in respiratory failure. Additionally, the weakness documented in all four limbs was especially acute and severely disabling. Finally, high HFGS and low MRC scores, both at nadir and at discharge, indicated marked increases in disease severity and poor short-term prognoses.

Electrophysiological investigations can provide an auxiliary diagnosis of GBS and are particularly useful for classifying GBS into the subgroups of acute inflammatory demyelinating polyneuropathy (AIDP), acute motor axonal neuropathy (AMAN), or acute motor sensory axonal neuropathy (AMSAN). In this study, electrophysiological abnormalities mainly affected motor nerve fibers but both terminal and proximal segments of the peripheral nervous system were also involved. Specifically, based on the electrophysiological criteria [8], five of the six patients were diagnosed with AMAN and one with AMSAN. All patients exhibited an axonal rather than demyelinating form of neuropathy, which predicted the severe clinical courses and poor outcomes that followed. Subsequently, we reviewed the electrophysiological features of post-traumatic GBS in the literature and found that after trauma, the axonal subtype of GBS is more common than the demyelinating subtype (Table 4). Yang et al. retrospectively analyzed 36 adult patients with GBS and found that the axonal subtype of GBS in post-trauma patients was proportionally higher than that in non-trauma patients, as seen in the present study [23]. The limited number of case reports of post-traumatic GBS in the literature does not support the conclusion that a causal relationship exists between the clinical phenotype and the history of trauma. It is not easy to affirm whether the co-existence of these two factors is anything more than mere coincidence.

**Table 4:** Descriptions of post-traumatic GBS in the academic literature

Author/Year	Number of case	Sex/number	Median age, years(range)	Antecedent events	Time from trauma to symptom onset (days)	EMG	Nerve biopsy
Rattananan et al. (2014) [1]	5	F/3	61 (35–68)	Surgery	within 30 days	Neuropathy with active denervation;	Perivascular inflammatory collections;increased axonal degeneration.
Staff et al. (2010) [2]	21	F/11	65 (24–83)	Surgery	within 30 days	Neuropathy with active denervation;	Increased epineurial perivascular inflammation;17 patients had increased axonal degeneration.
Huang et al. (2015) [3]	4	M/4	57 (50–69)	Spine Surgery:	within 1 week	Neuropathy and 2 cases with active denervation	not done
Scozzafava et al. (2008) [4]	1	M/1	28 (28)	Spinal cord injury	within 1 day	Severe axonal polyneuropathy	not done
Tan et al. (2010) [5]	1	M/1	44 (44)	Head injury	1 week	Neuropathy with active denervation;	Presence of lymphocytes and severe axonal degeneration.
Al-Hashel et al. (2013) [6]	2	F/1	39 (31–47)	Traumatic bone injury	within 1 week	1 with features of mixed axonal and demyelinating neuropathy	not done
Rivas et al. (2008) [7]	1	M/1	55 (55)	Head injury	1 week	An inexcitability of all nerves with active denervation;	A severe loss of myelinated axons without significant demyelination.

*GBS* Guillain-Barré syndrome, *F* female, *M* male, *EMG* electromyography

About half of the patients with GBS are positive for serum antibodies to various gangliosides, including GM1, GM1b, GM2, GD1a, GalNAc-GD1a, GD1b, GD2, GD3, GT1a, and GQ1b [24–26]. Previous studies suggest that most of these antibodies are specific for defined subgroups of GBS. For example, GM1, GD1a, GD1b, and GalNAc-GD1a antibodies are associated with axonal variants of GBS, whereas GD3, GT1a, and GQ1b antibodies are related to ophthalmoplegia and Miller-Fisher syndrome [27]. In this study, IgG anti-ganglioside antibodies were detected in four of the five patients tested. Patients #3 and #6, who experienced more serious muscle weakness and hypoesthesia were seropositive for GM1 and GD1b antibodies. This combination of serum-positive anti-ganglioside antibodies and electrophysiological abnormalities further illustrates that GBS with predominant axonal damage is most common after trauma.

The cases reported here highlight the importance of differentiating axonal GBS from critical illness polyneuropathy, which is a common cause of axonal polyneuropathy in trauma patients [19]. However, this can be difficult as axonal GBS can have striking similarities to critical illness polyneuropathy, in terms of clinical presentation and electrodiagnostic data. Cranial nerve involvement, such as that associated with bifacial weakness, and dysautonomia are uncommon in critical illness polyneuropathy. In these patients the degree of sensory symptoms and sensory nerve involvement tends to be mild. Albumino-cytological dissociation in CSF and the presence of certain serum anti-ganglioside antibodies also support a diagnosis of GBS. Finally, critical illness polyneuropathy does not generally respond to IVIG and/or plasma exchange, whereas GBS does. Despite these features, in the setting of critical illness or trauma, it remains a diagnostic challenge to distinguish axonal GBS from critical illness polyneuropathy.

Given the heterogeneity of the patients with post-traumatic GBS, it is postulated that the underlying mechanisms are based on a trauma-related disruption of the cellular and humoral immune system. Trauma often leads to transient immunosuppression and promotes clinical or subclinical exogenous infection [6, 28]. Immunosuppression could induce an alteration of immune tolerance and exogenous infection could elicit cross-reactive antibodies [3]. Together they could promote an autoimmune attack on peripheral nerves, resulting in the occurrence of axonal-type GBS. Conduction failure in the acute phase of axonal GBS could be attributed to lowered safety factors due to a dysfunction of the ion channels or due to microstructural changes at the nodes of Ranvier or paranodal regions caused by anti-ganglioside antibodies. The specific tissue distribution of these gangliosides in peripheral nerves could result in their characteristic clinical features. Therefore, the proliferation of serum antibodies against gangliosides shown here may represent an indirect trigger of GBS via a response to opportunistic infection rather the hypothesized direct incitement by trauma.

Post-traumatic GBS is a rapidly progressive and severe neurologic complication that occurs after trauma [15, 18, 19]. Thus, when there is unexplainable progressive muscle weakness after trauma, GBS should be taken into consideration and corresponding measures should be taken to relieve the condition. Both general medical care and immunological treatment are essential. All patients with sufficient suspicion of post-traumatic GBS should be monitored for possible respiratory failure and cardiac arrhythmia, and timely transfer to intensive care unit when needed. Reports of GBS in trauma patients is limited to case reports and no systematic research has been found so far discussing its immunological treatment. Therefore, an empiric course of intravenous immunoglobulin or plasma exchange might be valuable as it has been shown to improve prognosis [5, 18, 19]. Moreover, we found that some cases showed some clinical improvement, while others did not, when treated with intravenous methylprednisolone [15, 17]. Therefore, further research regarding the immunological treatment of post-traumatic GBS are required.

The limitations of our study include the relatively small sample size and the failure to identify pathogens. However, this first-ever reported case series of ganglioside-associated post-traumatic GBS may alert us to consider this diagnosis in patients with paralysis after trauma.

## Conclusions

The clinical presentations and laboratory findings described here played an important part in the diagnosis of post-traumatic GBS as likely immune-response-related nerve damage. The characteristic outcomes of the six patients studied were extremely severe disease, poor prognosis, and delayed recoveries. Such patients often have axonal damage. Therefore, electrophysiological investigations are important for the diagnosis and identification of different subtypes of GBS. This may facilitate early diagnosis and treatment to help prevent morbidity and improve prognosis.

## Abbreviations

AIDP: Acute inflammatory demyelinating polyneuropathy
AMAN: Acute motor axonal neuropathy
AMSAN: Acute motor sensory axonal neuropathy
CMAP: Compound muscle action potential
CSF: Cerebrospinal fluid
DL: Distal latency
ELISA: Enzyme-linked immunosorbent assay
GBS: Guillain-Barré syndrome
HFGS: Hughes Functional Grading Scale
MCV: Motor nerve conduction velocity
MRC: Medical Research Council
NCS: Nerve conduction study
SCV: Sensory nerve conduction velocity
SNAP: Sensory nerve action potential
